# Neutralizing Effects of Small Molecule Inhibitors and Metal Chelators on Coagulopathic *Viperinae* Snake Venom Toxins

**DOI:** 10.3390/biomedicines8090297

**Published:** 2020-08-20

**Authors:** Chunfang Xie, Laura-Oana Albulescu, Mátyás A. Bittenbinder, Govert W. Somsen, Freek J. Vonk, Nicholas R. Casewell, Jeroen Kool

**Affiliations:** 1 Amsterdam Institute of Molecular and Life Sciences, Division of BioAnalytical Chemistry, Department of Chemistry and Pharmaceutical Sciences, Faculty of Science, Vrije Universiteit Amsterdam, De Boelelaan 1085, 1081HV Amsterdam, The Netherlands; c.xie@vu.nl (C.X.); m.a.bittenbinder@vu.nl (M.A.B.); g.w.somsen@vu.nl (G.W.S.); freek.vonk@naturalis.nl (F.J.V.); 2 Centre for Analytical Sciences Amsterdam (CASA), 1098 XH Amsterdam, The Netherlands; 3 Centre for Snakebite Research and Interventions, Liverpool School of Tropical Medicine, Pembroke Place, Liverpool L3 5QA, UK; Laura-Oana.Albulescu@lstmed.ac.uk (L.-O.A.); Nicholas.Casewell@lstmed.ac.uk (N.R.C.); 4 Centre for Drugs and Diagnostics, Liverpool School of Tropical Medicine, Pembroke Place, Liverpool L3 5QA, UK; 5 Naturalis Biodiversity Center, 2333 CR Leiden, The Netherlands

**Keywords:** snakebite treatments, marimastat, varespladib, dimercaprol, DMPS, nanofractionation

## Abstract

Animal-derived antivenoms are the only specific therapies currently available for the treatment of snake envenoming, but these products have a number of limitations associated with their efficacy, safety and affordability for use in tropical snakebite victims. Small molecule drugs and drug candidates are regarded as promising alternatives for filling the critical therapeutic gap between snake envenoming and effective treatment. In this study, by using an advanced analytical technique that combines chromatography, mass spectrometry and bioassaying, we investigated the effect of several small molecule inhibitors that target phospholipase A_2_ (varespladib) and snake venom metalloproteinase (marimastat, dimercaprol and DMPS) toxin families on inhibiting the activities of coagulopathic toxins found in *Viperinae* snake venoms. The venoms of *Echis carinatus*, *Echis ocellatus*, *Daboia russelii* and *Bitis arietans*, which are known for their potent haemotoxicities, were fractionated in high resolution onto 384-well plates using liquid chromatography followed by coagulopathic bioassaying of the obtained fractions. Bioassay activities were correlated to parallel recorded mass spectrometric and proteomics data to assign the venom toxins responsible for coagulopathic activity and assess which of these toxins could be neutralized by the inhibitors under investigation. Our results showed that the phospholipase A_2_-inhibitor varespladib neutralized the vast majority of anticoagulation activities found across all of the tested snake venoms. Of the snake venom metalloproteinase inhibitors, marimastat demonstrated impressive neutralization of the procoagulation activities detected in all of the tested venoms, whereas dimercaprol and DMPS could only partially neutralize these activities at the doses tested. Our results provide additional support for the concept that combinations of small molecules, particularly the combination of varespladib with marimastat, serve as a drug-repurposing opportunity to develop new broad-spectrum inhibitor-based therapies for snakebite envenoming.

## 1. Introduction

Bites by venomous snakes cause 81,000–138,000 deaths per annum, with the majority occurring in the rural resource-poor regions of the tropics and sub-tropics [1]. The venomous snakes responsible for the vast majority of severe envenomings are members of the *Viperidae* and *Elapidae* families [2,3]. Elapid snakes have venoms that are highly abundant in neurotoxins that disable muscle contraction and cause neuromuscular paralysis [1,4]. Contrastingly, viper venoms typically contain numerous proteins that disrupt the functioning of the coagulation cascade, the hemostatic system and tissue integrity [4,5]. Envenomings caused by these snakes can cause prominent local effects including necrosis, hemorrhage, edema and pain, and often result in permanent disabilities in survivors [6,7]. One of the most common but serious pathological effects of systemic viper envenoming is coagulopathy, which renders snakebite victims vulnerable to suffering lethal internal hemorrhages [8]. Venom induced coagulopathy following bites by viperid snakes is predominately the result of the synergistic action of venom enzymes, such as phospholipases A_2_ (PLA_2_s), snake venom serine proteinases (SVSPs) and snake venom metalloproteinases (SVMPs) [9,10,11]. PLA_2_s can prevent blood clotting via anticoagulant effects. Enzymatic PLA_2_s function by hydrolyzing glycerophospholipids at the sn-2 position of the glycerol backbone releasing lysophospholipids and fatty acids [12]. SVSPs can proteolytically degrade fibrinogen and release bradykinins from plasma kininogens [13,14]. SVMPs act on various clotting factors to stimulate consumption coagulopathy and can also degrade capillary basement membranes, thereby increasing vascular permeability and causing leakage [10,15,16]. These toxins can therefore work synergistically to cause systemic hemorrhage and coagulopathy.

The only specific therapies currently available for treating snake envenoming are animal-derived antivenoms. Consisting of immunoglobulins purified from hyperimmunized ovine or equine plasma/serum, these products save thousands of lives each year, but are associated with a number of therapeutic challenges, including limited cross-snake species efficacies, poor safety profiles and, for many snakebite victims residing in remote rural areas in developing countries, unacceptable issues with affordability and accessibility [17]. Small molecule toxin inhibitors are regarded as promising candidates for the development of affordable broad-spectrum snakebite treatments, as these can block the enzymatic activities of venoms [18,19,20]. Varespladib, an indole-based nonspecific pan-secretory PLA_2_ inhibitor has been studied extensively for repurposing for snakebite. Having originally been investigated in Phase II and III clinical trials for treating septic shock, coronary heart disease and sickle cell disease-induced acute chest syndrome [21,22], varespladib has since been shown to be highly potent in suppressing venom-induced PLA_2_ activity, both in vitro and in vivo in murine models [23]. Varespladib shows great promise against neurotoxic elapid snake venoms and has been shown to prevent lethality in murine in vivo models of envenoming [24], but is seemingly also capable of inhibiting certain myotoxic and coagulotoxic symptoms induced by snake venoms [25,26]. Moreover, varespladib has been demonstrated to inhibit the anticoagulant activity of *Pseudechis australis* snake venom, which was not neutralized by its currently used antivenom [27].

A number of other small molecules have shown promise for repurposing to inhibit SVMP venom toxins. Marimastat is a broad-spectrum matrix metalloprotease inhibitor that functions by binding to the active site of matrix metalloproteinases where it coordinates the metal ion in the binding pocket [28,29]. As a water-soluble orally bioavailable matrix metalloproteinase inhibitor [30,31], marimastat reached phase II and III clinical trials for multiple solid tumor types [32,33,34], including pancreatic, lung, breast, colorectal, brain and prostate cancer [35,36,37]. SVMPs are toxins that are structurally and functionally homologous to matrix metalloproteinses [38,39,40]. Like other compounds in this class of drugs (e.g., batimastat [41]), marimastat is a promising drug candidate for treating snakebite due to its inhibitory capabilities against SVMP toxins [42,43]. Marimastat was found to effectively inhibit the hemorrhagic, coagulant and defibrinogenating effects and proteinase activities induced by *Echis ocellatus* venom [42]. Dimercaprol, a historical drug approved by the World Health Organization (WHO) for treatment of heavy metal poisoning [44], contains two metal-chelating thiol groups and has long been used against arsenic, mercury, gold, lead and antimony intoxication [45,46,47]. It also represents a treatment option for Wilson’s disease in which the body retains copper. Moreover, it has been studied as a candidate for acrolein detoxification as it can effectively reduce the acrolein concentration in vivo in murine because of its ability to bind to both the carbon double bond and aldehyde group of acrolein. The water-soluble, tissue-permeable and licensed metal chelator, 2,3-dimercaptopropane-1-sulfonic acid (DMPS), is also suitable for treating acute and chronic heavy metal intoxication including lead, mercury, cadmium and copper [48,49]. It was recently shown that both dimercaprol and DMPS displayed potential for repurposing as small molecule chelators to treat snake envenoming [20], most probably by chelating and removing Zn^2+^ from the active site of Zn^2+^-dependent SVMPs. Of the two drugs, DMPS showed highly promising preclinical efficacy when used as an early oral intervention after envenoming by the SVMP-rich venom of the West African saw-scaled viper (*Echis ocellatus*), prior to later antivenom treatment with antivenom [20]. In addition to protecting against venom-induced lethality, DMPS was also demonstrated to drastically reduce local venom-induced hemorrhage [20]. Thus, marimastat, dimercaprol and DMPS all represent promising candidates for drug repurposing as snakebite therapeutics, as they either inhibit SVMPs or chelate the Zn^2+^ ion required for SVMP catalysis.

Nanofractionation analytics, which is a recently developed high resolution and high throughput format of traditional bioassay-guided fractionation, is regarded as an effective method for screening complex bioactive mixtures such as venoms to rapidly identify and in parallel directly characterize separated venom toxins biochemically (i.e., for selected bioactivities), by combing reversed-phase liquid chromatography (RPLC) with parallel post-column bioassays, mass spectrometry (MS) and proteomics analysis [50,51,52]. In this paper, the coagulopathic properties of various snakes from the medically important viper subfamily *Viperinae* (*Echis carinatus*, *E. ocellatus*, *Daboia russelii* and *Bitis arietans*) were evaluated using nanofractionation analytics in combination with a high-throughput coagulation assay, and the inhibitory capabilities of varespladib, marimastat, dimercaprol and DMPS against the coagulopathic toxicities of the resulting snake venom fractions revealed. To this end, bioactivity chromatograms were acquired after fractionation, and parallel obtained mass spectrometry and proteomics data were used to correlate the observed bioactivities with the identity of the venom toxins responsible for the observed enzymatic effects. Thus, we assessed the ability of varespladib, marimastat, dimercaprol and DMPS to neutralize the coagulopathic venom components. The results indicated that varespladib in combination with heavy metal chelators and/or broad-spectrum protease inhibitors could be viable first line therapeutic candidates for initial and adjunct treatment of coagulopathic snakebite envenoming.

## 2. Experimental

### 2.1. Chemicals

Water from a Milli-Q Plus system (Millipore, Amsterdam, The Netherlands) was used. Acetronitrile (ACN) and formic acid (FA) were supplied by Biosolve (Valkenswaard, The Netherlands). Calcium chloride (CaCl_2_, dehydrate, ≥99%) was from Sigma-Aldrich (Zwijndrecht, The Netherlands) and was used to de-citrate plasma to initiate coagulation in the coagulation assay. Phosphate buffered saline (PBS) was prepared by dissolving PBS tablets (Sigma-Aldrich) in water according to the manufacturer’s instructions and was stored at −4 °C for no longer than one week prior to use. Sodium citrated bovine plasma was obtained from Biowest (Nuaillé, France) as sterile filtered. The plasma (500 mL bottle) was defrosted in a warm water bath, and then quickly transferred to 15 mL CentriStar^TM^ tubes (Corning Science, Reynosa, Mexico). These 15 mL tubes were then immediately re-frozen at −80 °C, where they were stored until use. Venoms were sourced from either wild-caught specimens maintained in, or historical venom samples stored in, the Herpetarium of the Liverpool School of Tropical Medicine (LSTM). This facility and its protocols for the expert husbandry of snakes are approved and inspected by the UK Home Office and the LSTM and University of Liverpool Animal Welfare and Ethical Review Boards. The venom pools were from vipers with diverse geographical localities, namely: *B. arietans* (Nigeria), *D. russelii* (Sri Lanka), *E. carinatus* (India) and *E. ocellatus* (Nigeria). Note that the Indian *E. carinatus* venom was collected from a single specimen that was inadvertently imported to the UK via a boat shipment of stone, and then rehoused at LSTM on the request of the UK Royal Society for the Prevention of Cruelty to Animals (RSPCA). Venom solutions were prepared by dissolving lyophilized venoms into water to a concentration of 5.0 ± 0.1 mg/mL and were stored at −80 °C until use. The compounds varespladib (A-001), marimastat ((2S,3R)-N4-[(1S)-2,2-Dimethyl-1-[(methylamino)carbonyl] propyl]-N1,2-dihydroxy-3-(2-methylpropyl) butanedia- mide), dimercaprol (2,3-Dimercapto-1-propanol) and DMPS (2,3-dimercapto-1-propane-sulfonic acid sodium salt monohydrate) were purchased from Sigma-Aldrich. They were dissolved in DMSO (≥99.9%, Sigma-Aldrich) to a concentration of 10 mM and stored at −20 °C. Prior to use, these four compounds were diluted in PBS buffer to the described concentrations.

### 2.2. Venom Nanofractionation

All venoms were nanofractionated onto transparent 384-well plates, and the plates with fractions were freeze dried overnight, according to the method described in our previous published papers by Slagboom et al. [53] and Xie et al. [52,54]. A detailed description can also be found in the Supporting Information (Section S1).

### 2.3. Plasma Coagulation Activity Assay

The HTS plasma coagulation assay used in this study was developed by Still et al. [55]. Sample preparation, assay performed and data analysis were described in our previous published paper by Still et al. [55], Slagboom et al. [53] and Xie et al. [52,54]. The final concentrations of the inhibitor solutions used in the coagulation bioassay were 20 μM, 4 μM and 0.8 μΜ, and in some cases 0.16 μM, 0.032 μM and 0.0064 μM. A detailed description can also be found in the Supporting Information (Section S2).

### 2.4. Correlation of Biological Data with MS Data

The corresponding accurate mass(es) and proteomics data for each venom fraction in this study have already been acquired by Slagboom et al. [53] and as such were correlated with the bioactivity chromatograms obtained in the current study. For venoms under study in this project that were not studied by Slagboom et al. [53], the same procedure as previously described [53] was followed to acquire and process proteomics data on these snake venoms. The UniprotKB database was used to determine the toxin class and any known functions for the relevant toxins thought to be responsible for the observed coagulopathic toxicities. For LC separations performed at different times and in different labs, the retention times of eluting snake venom toxins may differ slightly. The LC-UV chromatograms (measured at 220 nm, 254 nm and 280 nm), which provided characteristic fingerprint profiles for each venom fraction, were used to negotiate these retention time shifts. By using the LC-UV data, the chromatographic bioassay data from this study was correlated with the MS total-ion currents (TICs), extracted-ion chromatograms (XICs), and proteomics data obtained by Slagboom et al. [53]. In order to construct useful XICs, MS spectra were extracted from the time frames that correlated with regions in the chromatograms for each bioactive peak. Then, for all *m/z* values showing a significant signal observed in the mass spectra, XICs were plotted. In turn, these XICs were used for matching with peak retention times of bioactive compounds in the chromatograms. The exact masses matching the bioactives were tentatively assigned based on matching peak shape and correlation with retention times in bioassay traces. More specifically, the *m/z*-values in the MS data were correlated to each bioactive peak using the accurate monoisotopic masses determined by applying the deconvolution option in the MS software. For the proteomics data, in-well tryptic digestions were previously performed by Slagboom et al. [53] on snake venom fractions. These proteomics results were directly correlated to the coagulopathic activities that were indicated by the bioassay chromatograms.

## 3. Results

In this study, a nanofractionation approach was used to evaluate the inhibitory effects of varespladib, marimastat, dimercaprol and DMPS on the coagulopathic properties of venom toxins fractionated from a variety of *Viperinae* snake species. A recently developed low-volume HTS coagulation bioassay was used to assess the coagulation activities of LC-fractionated venoms in a 384-well plate format. These coagulopathic activities were correlated to parallel obtained MS and proteomics data to determine which specific venom toxins were neutralized by the potential inhibitors. All analyses were performed in at least duplicate to ensure reproducibility, and used venom concentrations of 1.0 mg/mL.

### 3.1. Inhibitory Effects of Varespladib, Marimastat, Dimercaprol and DMPS on Echis Venoms

**Figure 1:**
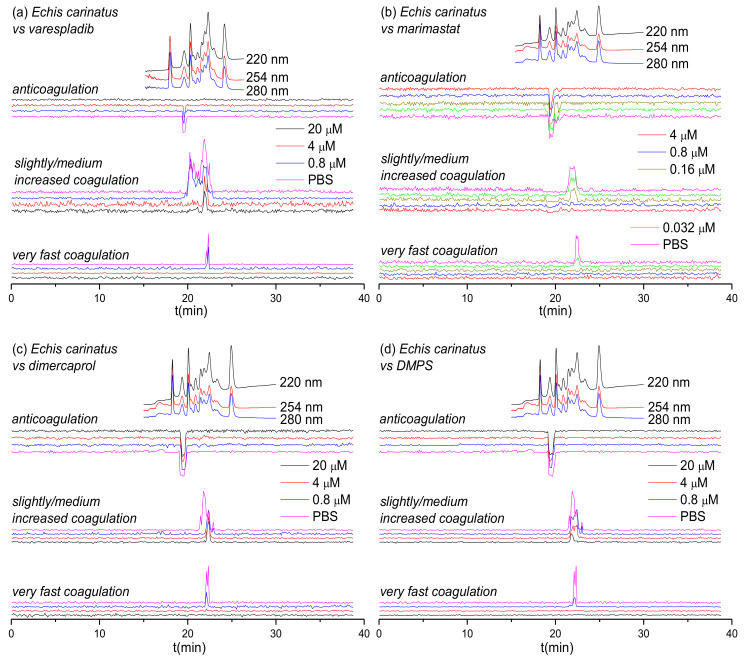
UV absorbance chromatograms and reconstructed coagulopathic toxicity chromatograms of nanofractionated toxins from *E. carinatus* venom in the presence of different concentrations of (**a**) varespladib, (**b**) marimastat, (**c**) dimercaprol and (**d**) DMPS.

**Figure 2:**
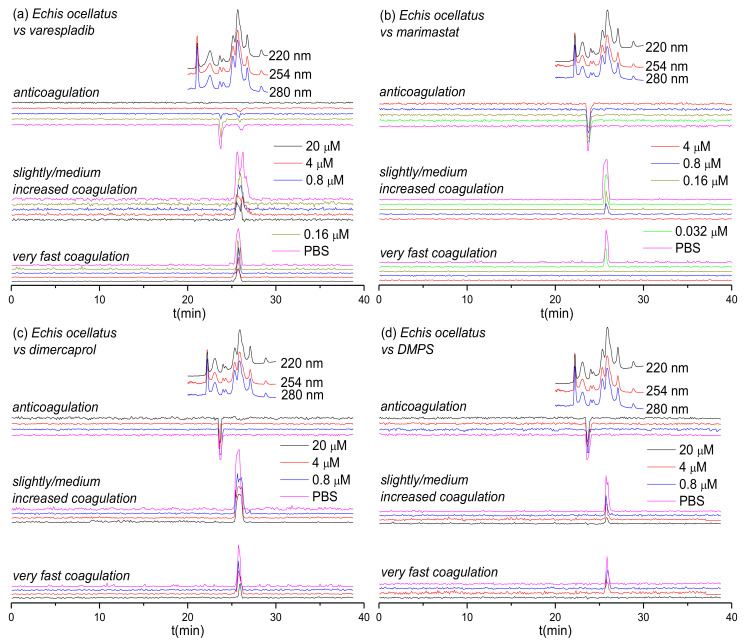
UV absorbance chromatograms reconstructed coagulopathic toxicity chromatograms of nanofractionated toxins from *E. ocellatus* venom in the presence of different concentrations of (**a**) varespladib, (**b**) marimastat, (**c**) dimercaprol and (**d**) DMPS.

Two geographically distinct saw-scaled viper venoms (genus *Echis*) were investigated in this study, specifically from the Indian species *E. carinatus* and the west African species *E. ocellatus*. The inhibitory effects of varespladib, marimastat, dimercaprol and DMPS against the coagulopathic activities observed for LC fractions of both venoms were investigated in a concentration-dependent fashion (Figure 1 and Figure 2). Duplicate bioassay chromatograms together with a detailed description of each coagulopathic peak observed are presented in the Supporting Information (Section S3, Figures S1–S8).

Figure 1 shows the bioassay chromatograms of nanofractionated venom toxins from *E. carinatus* in the presence of different concentrations of varespladib, marimastat, dimercaprol and DMPS. In the venom-only analysis, potent procoagulation activities were observed in the very fast coagulation chromatogram (22.0–22.9 min) and the slightly/medium increased coagulation chromatogram (21.2–23.1 min and/or 19.9–21.2 min), while anticoagulation activities were observed in the anticoagulation chromatogram (19.1–19.9 min). Interestingly, the PLA_2_-inhibitor varespladib inhibited both the anticoagulation and procoagulation activities, with the exception of one major peak observed in the slightly/medium increased coagulation chromatogram. In contrast, marimastat, dimercaprol and DMPS only exerted inhibitory effects on the procoagulation activities of *E. carinatus* venom. The anticoagulation activity of *E. carinatus* venom was fully inhibited by varespladib at a 20 μM concentration, while the very fast procoagulation activity was fully inhibited by varespladib, dimercaprol and DMPS at a concentration of 4 μM. Marimastat superseded the other small molecules by fully inhibiting the very fast procoagulation activity at a concentration of 0.16 μM. The slightly/medium increased coagulation activity was fully inhibited by 0.8 μM marimastat, but a sharp positive peak (21.7–22.2 min) was still retained following incubation with 20 μM varespladib. Dimercaprol only inhibited the front peak (21.3–22.1 min) present in the slightly/medium increased coagulation activity chromatogram, while DMPS inhibited mostly the tailing part (22.2–23.1 min) of this peak at its highest concentration tested (20 μM). Overall, DMPS was found to be more effective than dimercaprol in abrogating the procoagulation toxicities of *E. carinatus* venom. These findings demonstrate that the tested inhibitors have different specificities, but that marimastat most effectively inhibits the procoagulant components, and varespladib the anticoagulant components, of *E. carinatus* venom.

Figure 2 shows the bioassay chromatograms for nanofractionated toxins from *E. ocellatus* venom in the presence of different concentrations of varespladib, marimastat, dimercaprol and DMPS. In the venom-only analysis, we observed similar results to those obtained for *E. carinatus* venom; multiple co-eluting sharp peaks were present in the very fast coagulation chromatogram (25.1–26.2 min), the slightly/medium increased coagulation chromatogram (25.1–27.1 min) and the anticoagulation chromatogram (23.4–24.4 min). All peaks decreased in height and width with increasing varespladib concentrations. The potent negative peak (23.4–24.4 min) in the anticoagulation chromatograms was fully inhibited by 4 μM varespladib and the later eluting weakly negative peak (25.9 min) by 20 μM varespladib. While full inhibition of anticoagulation activities was achieved, the procoagulation activities were not fully inactivated at the highest varespladib concentration tested (20 μM). However, both the very fast coagulation activity and the slightly/medium increased coagulation activity were also somewhat reduced by varespladib in a concentration-dependent fashion. Similar findings, whereby both very fast and slightly/medium increased coagulation were reduced in a concentration dependent manner but not fully abrogated, were also observed for dimercaprol, although this inhibitor had no effect on anticoagulant venom activities. Marimastat and DMPS also had no effect on anticoagulant venom activity, but effectively inhibited the procoagulant actions of *E. ocellatus* venom. Very fast procoagulation activity was fully inhibited at a lower concentration of marimastat (0.16 μM) than DMPS (20 μM), while slightly/medium increased coagulation activity was fully inhibited by 4 μM marimastat compared with almost complete inhibition observed when using 20 μM DMPS. Thus, similar to findings with *E. carinatus*, marimastat exhibited superior inhibition of procoagulant venom activities, while varespladib was the only inhibitor capable of abrogating anticoagulant venom effects.

### 3.2. Inhibitory Effect of Varespladib, Marimastat, Dimercaprol and DMPS on Daboia russelii Venom

**Figure 3:**
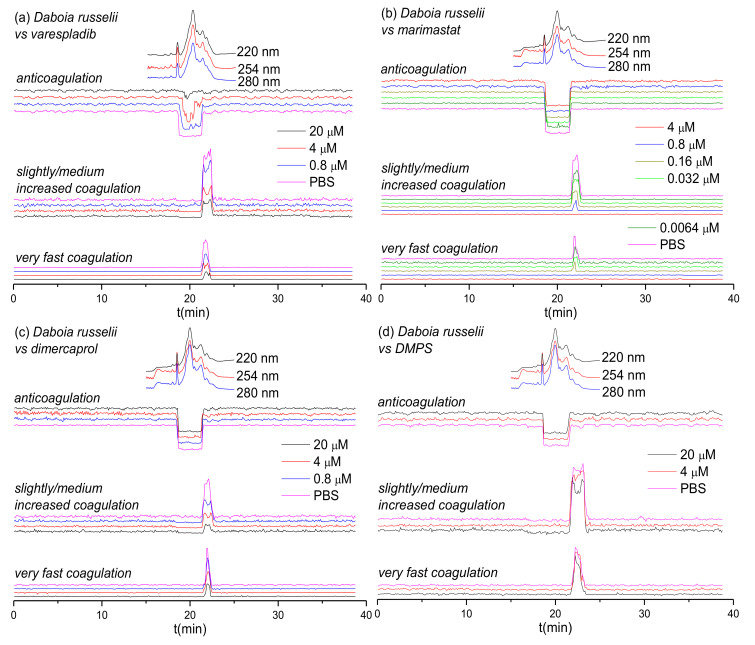
UV absorbance chromatograms and reconstructed coagulopathic toxicity chromatograms of nanofractionated toxins from *D. russelii* venom in the presence of different concentrations of (**a**) varespladib, (**b**) marimastat, (**c**) dimercaprol and (**d**) DMPS.

Next, we assessed the inhibitory capability of the same small molecule toxin inhibitors on a *Viperinae* snake from a different genus: the Russell’s viper (*Daboia russelii*), which is a medically-important species found in south Asia [56,57,58]. The inhibitory effects of varespladib, marimastat, dimercaprol and DMPS on the venom of *D. russelii* are shown in Figure 3. Duplicate bioassay chromatograms for *D. russelii* venom analyses can be found in the Supporting Information in Section S4 (Figures S9–S12). For the venom-only analysis, a strong positive peak was observed for both the very fast coagulation activity (21.5–22.4 min) and for the slightly/medium increased coagulation activity (21.5–22.8 min). A very broad and strong negative activity peak (18.6–21.5 min) was also observed, demonstrating potent anticoagulation activity. In terms of procoagulant venom effects, both very fast and slightly/medium increased coagulation activities decreased dose-dependently in the presence of varespladib, marimastat and dimercaprol, although neither varespladib nor dimercaprol could fully neutralize these activities. However, in line with the earlier findings for the two *Echis* spp., full neutralization of both types of procoagulation were observed with marimastat, at 0.8 μM for very fast coagulation activity and at 4 μM for slightly/medium increased coagulation activity. As anticipated, and again in line with findings observed with *Echis* spp., neither of the SVMP-inhibitors (marimastat and dimercaprol) abrogated anticoagulant venom activity. In contrast, varespladib showed potent inhibition of anticoagulation, as the broad and potent negative peak (18.6–21.5 min) decreased to only a very minor negative peak (19.5–20.2 min; 20 μM varespladib) with increasing varespladib concentrations. DMPS showed no inhibition on both the procoagulant and anticoagulant venom activities of *D. russelii* at the tested inhibitor concentrations of 20 μM and 4 μM.

### 3.3. Inhibitory Effects of Varespladib and Marimastat on Bitis arietans Venom

**Figure 4:**
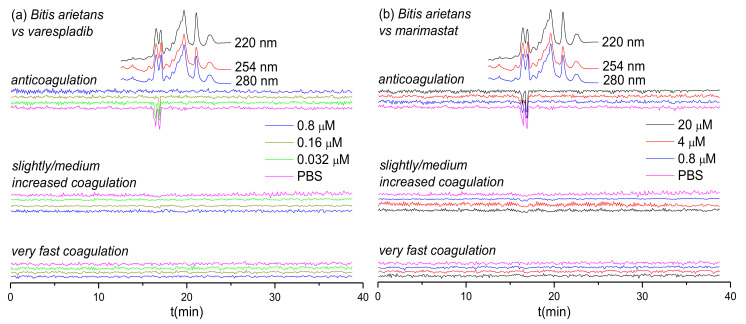
UV absorbance chromatograms and reconstructed coagulopathic toxicity chromatograms of nanofractionated toxins from *B. arietans* venom in the presence of different concentrations of (**a**) varespladib and (**b**) marimastat.

The inhibitory effects of varespladib and marimastat on the coagulopathic properties of venom of the puff adder (*B. arietans*), which is found widely distributed across sub-Saharan Africa and parts of the Middle East, are shown in Figure 4. Duplicate bioassay chromatograms for the *B. arietans* venom analyses are shown in the Supporting Information in Section S5 (Figures S13 and S14). In the venom-only analyses, anticoagulation activity was observed as two sharp negative peaks in the bioactivity chromatograms (16.2–16.7 min and 16.7–17.1 min); however, no procoagulation activity was detected, which is consistent with previous findings using this venom [59]. Consequently, of the three SVMP-inhibitors used elsewhere in this study, we only selected marimastat for assessment of toxin inhibition as a control for the PLA_2_-inhibitor varespladib. In line with findings from the other *Viperinae* species under study, increasing concentrations of varespladib resulted in full inhibition of the two negative anticoagulation peaks, at concentrations of 0.16 µΜ and 0.8 µΜ, respectively. Conversely, and also in line with our earlier findings, no anticoagulant inhibitory effects were observed with marimastat, even at concentrations of 20 µΜ.

### 3.4. Identification of Coagulopathic Venom Toxins Neutralized by Small Molecule Inhibitors

The MS and proteomics data previously obtained by Slagboom et al. [53] was next used to assign the venom toxins responsible for the observed coagulation activities tentative identifications. The resulting identifications are listed in Table 1. In addition, all tentatively identified anticoagulant PLA_2_s, including those found in our study not previously described as possessing anticoagulant properties in the UniprotKB database, are also provided in Table 1. For those toxins for which no exact mass data could be acquired by LC-MS, only the proteomics mass data retrieved from Mascot searches are presented in the table.

Based on the results from Figure 1, Figure 2, Figure 3 and Figure 4 and Table 1, the inhibitory effects of varespladib, marimastat, dimercaprol and DMPS on individual *Viperinae* venom toxins were assessed. PLA_2_ toxins were identified as toxin components responsible for anticoagulation in all species studied, except for *B. arietans*, for which C-Type Lectins (CTLs) were instead identified. All these identified anticoagulant toxins were fully abrogated by varespladib at various concentrations, as indicated in Table 1. Although the CTLs identified from *B. arietans* venom are highly likely to have co-eluted with other venom proteins, no other anticoagulants were identified from this venom. The toxins identified from the Mascot results for the procoagulant peaks of *E. ocellatus* venom included both SVMPs and CTLs. All these identified toxins were fully abrogated by marimastat at 0.16 μM or by DMPS at 20 μM concentrations. No procoagulant toxins could be identified from the Mascot results for *E. carinatus* and *D. russelii* venoms, but given that these bioactivity peaks were fully inhibited by marimastat at low concentrations, it seems reasonable to speculate that the procoagulant toxins responsible for these activities are mainly SVMPs. A detailed description of the results discussed here is provided in the Supporting Information in Section S6.

There are a number of challenges associated with interpreting the data presented here. In cases where multiple toxins elute closely, unambiguously assigning single toxins to each detected bioactivity is challenging. For bioactive compounds that eluted in activity peaks that were only partly inhibited, it is difficult to critically determine which of them was abrogated. This would require further improving LC separations under toxin non-denaturating and MS compatible eluent conditions. As a critical note, it is important to stress that despite venom toxins generally being stable, during chromatography within the nanofractionation analytics pipeline some venom toxins might have (partly) denatured and thereby lost their activity. 

## 4. Discussion

There is an urgent need for stable, effective and affordable snakebite treatments that can be administered in the field and in rural areas where medical access is limited. Small molecule inhibitors that specifically target a number of key classes of snake venom toxins have recently gained interest as candidates for therapeutic alternatives to conventional antivenom.

This study used a high-throughput screening assay combined with LC fractionation and parallel MS and proteomics data to assess the neutralizing capabilities of a selected number of small molecule inhibitors and chelators (i.e., varespladib, marimastat, dimercaprol and DMPS). The results of this study show that these compounds are capable of neutralizing the coagulopathic activities of individual toxins present in the venoms of a number of *Viperinae* species. While this is consistent with previous work on small molecule inhibitors and chelators that exhibit anti-hemorrhagic and anti-procoagulant activities of snake venoms [9,20,23,42,43,60], here we have studied the relative neutralization potencies of these small molecules on individual coagulopathic venom toxins. Our findings reveal that varespladib is not only effective against the activity of anti-coagulant PLA_2_ toxins, but also shows some inhibitory activity against procoagulant venom toxins. Varespladib potently and completely inhibited the anticoagulant activities detected in all venoms, except for *D. russelii*, for which almost complete inhibition was observed. Furthermore, varespladib showed some degree of inhibition against procoagulant venom activities across the various venoms, despite these activities not known to be mediated by PLA_2_ toxins. Contrastingly, of the SVMP-inhibitors tested, we demonstrate that their specificities are restricted to effects on procoagulant venoms toxins, and that the peptidomimetic hydroxamate inhibitor marimastat outperforms the metal chelators DMPS and dimercaprol in terms of potency. Marimastat potently inhibited procoagulant activities across the venoms tested and was unsurprisingly ineffective against anticoagulant venom activities. However, only moderate inhibition was observed for most venoms with the metal chelators, and no inhibition was found at all for DMPS on *D. russelii* venom. Neither DMPS nor dimercaprol inhibited the non-SVMP stimulated anticoagulant venom activities observed across the venoms.

The advantages of repurposing licensed medicines (e.g., DMPS and dimercaprol) or phase II-approved drug candidates (e.g., marimastat and varespladib) are that these molecules have demonstrated safety profiles and thus drug development times could be significantly shortened as these agents have extensive pharmacokinetic, bioavailability and tolerance data already associated with them [23,61,62]. The small size of these compounds, compared with conventional antibodies, confer desirable drug-favorable properties enabling rapid and effective tissue penetration and, depending on the pharmacokinetics and physicochemical properties of specific inhibitors, often make them amenable for oral delivery [61,63,64]. Indeed, both varespladib and DMPS have already been demonstrated to confer preclinical efficacy against snakebite via the oral route [20,63,64]. DMPS is readily absorbed following oral administration in humans, making it a strong candidate for an oral community-based therapy [65]; and there are no major side effects or teratogenic effects that have been reported on DMPS in murine models [66,67]. In contrast, dimercaprol is challenging for clinical use as it currently requires administration by painful intramuscular injection. Dosing regimens of each inhibitor therefore need further robust investigation in the context of snakebite in order to help inform lead candidate selection.

Certain small molecule inhibitors have been demonstrated to exhibit broad inhibition of specific toxin families across diverse medically-important snake species [23,59], as also evidenced here for the various coagulopathic toxins found across *Viperinae* venoms. However, these compounds typically target only a single family of enzymatic toxins (although varespladib seems perhaps capable of targeting more than one family; see Figure 1a, Figure 2a and Figure 3a), thus presenting a challenge for these molecules to become standalone therapeutics, as other non-inhibited toxins seem likely to still cause pathology in snakebite victims. It is therefore more likely that small molecule inhibitors will need to be combined into therapeutic mixtures, either with other toxin inhibitors or monoclonal antibodies, to generate snakebite therapeutics capable of neutralizing the most important pathological snake venom toxin families [26,59,61,62]. Small molecule inhibitors could serve as valuable prehospital snakebite treatments to delay the onset of severe envenoming before the arrival of victims to secondary or tertiary healthcare facilities to receive subsequent therapy (i.e., conventional antivenoms). This is important, because treatment delays are known to have major detrimental impacts on patient outcomes following snakebite [68,69]. Indeed, compounds such as varespladib and DMPS are already being explored in this regard [20,63,64], as they represent promising candidates to be used as bridging therapies for delaying the major effects of envenomation, and reducing the long time it typically takes rural, isolated, impoverished snakebite victims to receive any form of treatment.

The many limitations associated with conventional snakebite treatments have resulted in their weak demand, low availability and poor affordability, despite huge unmet medical need. Small molecule “toxin inhibitors” offer great potential to rapidly deliver inexpensive, safe and efficacious interventions in the community soon after a snakebite, prior to subsequent admission to a healthcare facility. Many small molecule inhibitors also offer superior pharmacokinetics in terms of efficiently reaching local tissues, such as bite sites, in contrary to antibodies. The findings presented here further support the exploration of such inhibitors as potential future snakebite treatments.

## 5. Conclusions

In this study, a recently developed HTS coagulation assay was combined with LC fractionation and parallel obtained MS and proteomics data to assess the neutralizing potency of several small molecule inhibitors and chelators (i.e., varespladib, marimastat, dimercaprol and DMPS) against the coagulopathic activities of individual toxins found in the venoms of *Viperinae* snakes. These compounds show great promise for the development of affordable, broad-spectrum, first-aid and clinical treatment of snakebite. Our data further strengthens recent findings suggesting that small molecule inhibitors, such as varespladib and marimastat, may have broad, cross-species, neutralizing capabilities that make them highly amenable for translation into new "generic” snakebite therapeutics. Given our evidence that both inhibitors have different specificities, our findings further support the concept that a therapeutic combination consisting of both of these Phase II-approved small molecule toxin inhibitors [59] show potential as a new broad-spectrum snakebite treatment.

## Figures and Tables

**Table 1:** Correlated MS and proteomics data for associated coagulopathic venom toxins. (Peak retention times are adapted from Figure 1, Figure 2, Figure 3 and Figure 4; PLA_2_ = phospholipase A_2_; SVMP = Snake Venom Metalloproteinase; CTL = C-Type Lectin).

Species	Peak Retention Time (min)	Mascot Results Matching the Exact Mass	Exact Mass from MS Data	Exact Mass from Mascot Data	Toxin Class	Activity	Dose Required for Full Inhibition
*E. carinatus*	19.1–19.9	PA2A1_ECHCA	–	16310	PLA_2_	Anticoagulant	20 μM varespladib
	19.9–23.1	–	–	–	–	Procoagulant	0.8 μM marimastat
*E. ocellatus*	23.4–24.4	PA2A5_ECHOC	13856.138	13856	PLA_2_	Anticoagulant	4 μM varespladib
	25.1–27.1	VM3E2_ECHOC	–	69426	SVMP	Procoagulant	0.16 μM marimastat/20 μM DMPS
	25.1–27.1	VM3E6_ECHOC	–	57658	SVMP	Procoagulant	0.16 μM marimastat/20 μM DMPS
	25.1–27.1	SL1_ECHOC	–	16601	CTL	Procoagulant	0.16 μM marimastat/20 μM DMPS
	25.1–27.1	SL124_ECHOC	–	16882	CTL	Procoagulant	0.16 μM marimastat/20 μM DMPS
*D. russelii*	18.6–21.5	PA2B8_DABRR	13587.225	13587	PLA_2_	Anticoagulant	20 μM varespladib
	18.6–21.5	PA2B5_DABRR	–	13587	PLA_2_	Anticoagulant	20 μM varespladib
	18.6–21.5	PA2B3_DABRR	–	13687	PLA_2_	Anticoagulant	20 μM varespladib
	21.5–22.8	–	–	–	–	Procoagulant	4 μM marimastat
*B. arietans*	16.7–17.1	SLA_BITAR	–	14935	CTL	Anticoagulant	0.8 μM varespladib
	16.7–17.1	SLB_BITAR	–	14798	CTL	Anticoagulant	0.8 μM varespladib

## Supplementary Materials

The supporting information related to this article can be found in “*Supplementary Materials: Neutralizing effects of small molecule inhibitors and metal chelators on coagulopathic Viperinae snake venom toxins*”. The following are available online at https://www.mdpi.com/2227-9059/8/9/297/s1, Figure S1: Duplicate bioassay chromatograms of nanofractionated *E. carinatus* venom in the presence of different concentrations of varespladib; Figure S2: Duplicate bioassay chromatograms of nanofractionated *E. carinatus* venom in the presence of different concentrations of marimastat; Figure S3: Duplicate bioassay chromatograms of nanofractionated *E. carinatus* venom in the presence of different concentrations of dimercaprol; Figure S4: Duplicate bioassay chromatograms of nanofractionated *E. carinatus* venom in the presence of different concentrations of DMPS; Figure S5: Duplicate bioassay chromatograms of nanofractionated *E. ocellatus* venom in the presence of different concentrations of varespladib; Figure S6: Duplicate bioassay chromatograms of nanofractionated *E. ocellatus* venom in the presence of different concentrations of marimastat; Figure S7: Duplicate bioassay chromatograms of nanofractionated *E. ocellatus* venom in the presence of different concentrations of dimercaprol; Figure S8: Duplicate bioassay chromatograms of nanofractionated *E. ocellatus* venom in the presence of different concentrations of DMPS; Figure S9: Duplicate bioassay chromatograms of nanofractionated *D. russelii* venom in the presence of different concentrations of varespladib; Figure S10: Duplicate bioassay chromatograms of nanofractionated *D. russelii* venom in the presence of different concentrations of marimastat; Figure S11: Duplicate bioassay chromatograms of nanofarctionated *D. russelii* venom in the presence of different concentrations of dimercaprol; Figure S12: Duplicate bioassay chromatograms of nanofarctionated *D. russelii* venom in the presence of different concentrations of DMPS; Figure S13: Duplicate bioassay chromatograms of nanofractionated *B. arietans* venom in the presence of different concentrations of varespladib; Figure S14: Duplicate bioassay chromatograms of nanofractionated *B. arietans* venom in the presence of different concentrations of marimastat.
